# Continental-scale patterns of hyper-cryptic diversity within the freshwater model taxon *Gammarus fossarum* (Crustacea, Amphipoda)

**DOI:** 10.1038/s41598-020-73739-0

**Published:** 2020-10-06

**Authors:** Remi Wattier, Tomasz Mamos, Denis Copilaş-Ciocianu, Mišel Jelić, Anthony Ollivier, Arnaud Chaumot, Michael Danger, Vincent Felten, Christophe Piscart, Krešimir Žganec, Tomasz Rewicz, Anna Wysocka, Thierry Rigaud, Michał Grabowski

**Affiliations:** 1 UMR CNRS 6282 Biogéosciences, Université Bourgogne Franche Comté, Dijon, France; 2 Department of Invertebrate Zoology and Hydrobiology, University of Lodz, Lodz, Poland; 3 Zoological Institute, University of Basel, Basel, Switzerland; 4 Institute of Ecology, Nature Research Centre, Vilnius Nature Research Centre, Institute of Ecology, Vilnius, Lithuania; 5 Department of Natural Sciences, Varaždin City Museum, Varaždin, Croatia; 6 Laboratoire d’écotoxicologie, INRAE, UR RiverLy, Villeurbanne, France; 7 UMR CNRS 73602 LIEC, Université de Lorraine, Metz, France; 8 Univ Rennes, CNRS, ECOBIO-UMR 6553, 35000 Rennes, France; 9 Department of Teacher Education Studies in Gospić, University of Zadar, Gospić, Croatia; 10 University of Guelph, Centre for Biodiversity Genomics, Guelph, ON Canada; 11 Department of Genetics and Biosystematics, University of Gdansk, Gdansk, Poland

**Keywords:** Biodiversity, Biogeography, Molecular ecology

## Abstract

Traditional morphological diagnoses of taxonomic status remain widely used while an increasing number of studies show that one morphospecies might hide cryptic diversity, i.e. lineages with unexpectedly high molecular divergence. This hidden diversity can reach even tens of lineages, i.e. hyper cryptic diversity. Even well-studied model-organisms may exhibit overlooked cryptic diversity. Such is the case of the freshwater crustacean amphipod model taxon *Gammarus fossarum*. It is extensively used in both applied and basic types of research, including biodiversity assessments, ecotoxicology and evolutionary ecology. Based on COI barcodes of 4926 individuals from 498 sampling sites in 19 European countries, the present paper shows (1) hyper cryptic diversity, ranging from 84 to 152 Molecular Operational Taxonomic Units, (2) ancient diversification starting already 26 Mya in the Oligocene, and (3) high level of lineage syntopy. Even if hyper cryptic diversity was already documented in *G. fossarum*, the present study increases its extent fourfold, providing a first continental-scale insight into its geographical distribution and establishes several diversification hotspots, notably south-eastern and central Europe. The challenges of recording hyper cryptic diversity in the future are also discussed.

## Introduction

In many areas of biology, including biodiversity assessments, eco-toxicology, environmental monitoring, and behavioural ecology, the species status of the studied organisms relies only on a traditional morphological definition^1^. However, a continually increasing number of studies of plants and animals show that one morphospecies might hide lineages with molecular divergences far exceeding what is expected at the intra-specific level in a given taxon^2,3^. This hidden diversity, nicknamed cryptic diversity (CD), can reach even tens of lineages for one morphospecies, i.e. showing hyper cryptic diversity (HCD)^1^. Even well-studied model-organisms may exhibit an overlooked CD^4^. This hidden diversity occurs at various geographic scales, and it has often been revealed to have pre-Pleistocene origins^5^.

Cryptic diversity has only recently been taken into account in both basic and applied ecology, as well as in evolutionary biology^6–8^. Even basic evolutionary studies were shown to be impacted by CD if two or more cryptic species are merged into one analysis. Such is the case for sexual selection analyses^9^, studies upon host-parasite co-evolution^6,10,11^, functional ecology^12,13^ or population genetics^14^, and is likely to impact genome assembly, even in the case of model organisms. Similarly, results of biodiversity assessments may be severely underestimated if ignoring the presence of CD^15^. At the macroecological scale, considering CD often generates a more nuanced view on determinants of biodiversity patterns^8^, and it should be taken into account while defining conservation units, especially in case of endemic cryptic lineages^16,17^. Simulations of distributional patterns under future climatic global change underestimate the real scale of biodiversity loss when CD is not taken into account^18^. Finally, ecotoxicological and biomonitoring studies are at risk of being flawed, as some authors reported that different cryptic species might differ in their tolerance to contaminants and ecosystem deterioration^19–23^.

While CD was detected across a wide range of taxa^3^, some may be more prone than others^2^. As a rule of thumb, morphospecies with broad geographic distributions, living in patchy habitats and having limited dispersal abilities tend to contain more divergent lineages^2,24–26^. For example, many freshwater macroinvertebrates^27^ and especially amphipod crustaceans are known to be associated with a high level of CD^5,19,28–39^.

**Figure 1:**
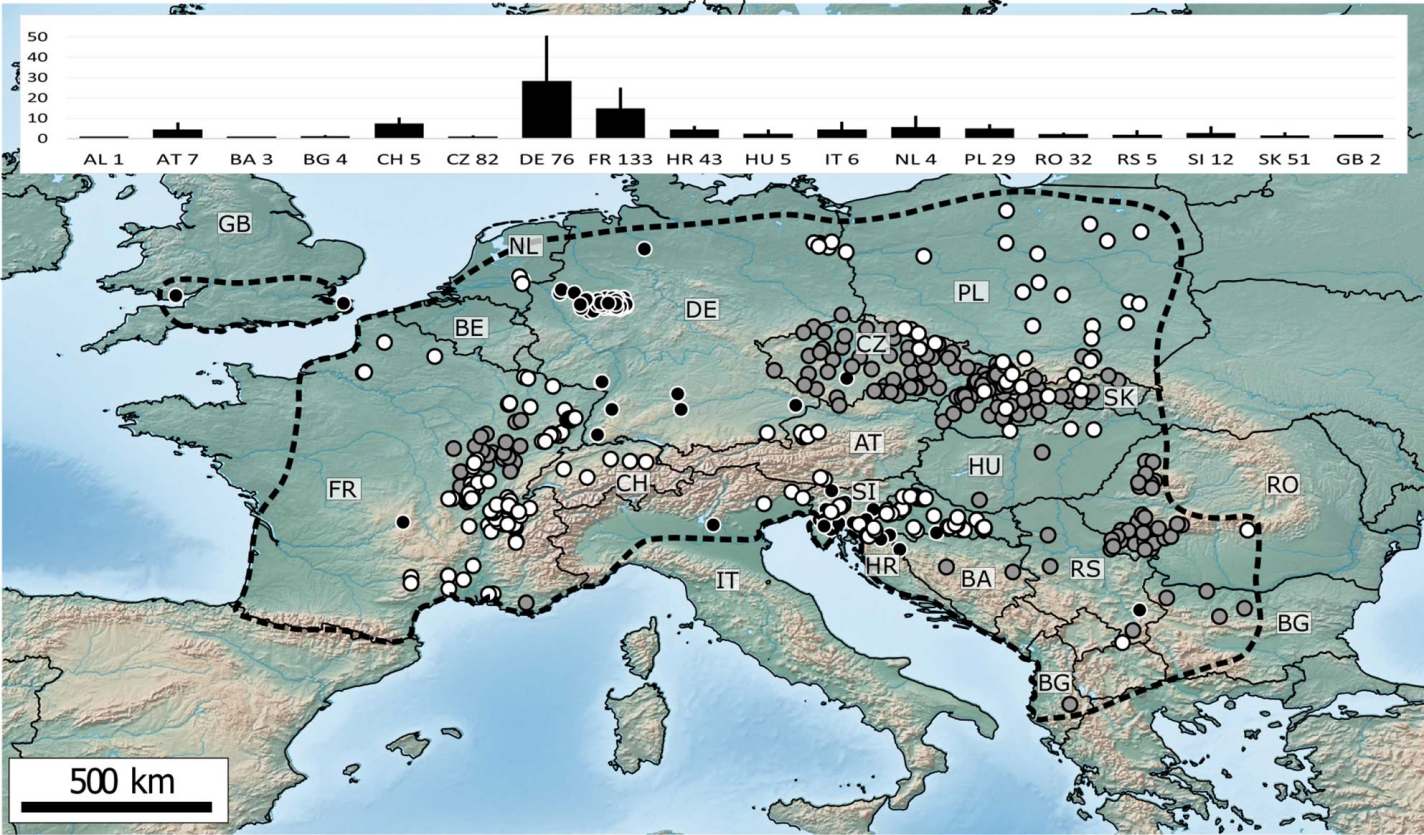
Map of *Gammarus fossarum* sampling sites. Dots indicate sampling sites (498); white with black contour represent new original data (206 sites), grey with black contour indicate sites associated with authors' previous publications (198 sites), black with white contour indicate sites for data derived from other published papers (94 sites). To prevent overlap of very closely located sites, some positions are slightly shifted. Thin blue-grey lines correspond to first-order river, and black lines are country borders. Dashed grey line represents the simplified distribution range limits for *G. fossarum*, adapted from Piscart and Bollache^96^. Countries (19) are indicated by their corresponding two-letter ISO codes: AL, Albania; AT, Austria; BA, Bosnia and Herzegovina; BE, Belgium; BG, Bulgaria; CH, Switzerland; CZ, Czechia; DE, Germany; FR, France; HR, Croatia; HU, Hungary; IT, Italy; NL, Netherlands; PL, Poland; RO, Romania; RS, Serbia; SI, Slovenia; SK, Slovakia; UK, United Kingdom. Top Inset represent sampling effort for each country: mean number of samples per site + S.E.. Number by the ISO code is the number of sites sampled. Map created with QGIS 3.4.5 (https://www.qgis.org/fr/site/).

Here, we focus on the freshwater amphipod morphospecies *Gammarus fossarum* Koch, 1836, widespread in continental Europe and recently identified in the United Kingdom^40^ (Fig. 1). The species is mostly epigean, being present in a wide range of habitats, but sometimes is found even in shallow groundwaters. It is a keystone species in streams and rivers, where it occurs in high densities and plays a major role in the food web as the primary scavenger/shredder of organic matter as well as a food source for fish and macroinvertebrates (e.g.^41,42^). Thus, it is commonly used as a sentinel species in freshwater risk assessment of ecosystem quality, especially in the frame of the European Union's Water Framework Directive (EU WFD)^43^. It is also used in eco-physiology, e.g. in association with global warming studies^44,45^, as well as, extensively, in eco-toxicology^46–49^. This species has also been emerging as a model for molecular reproduction physiology^50^. Finally, *G. fossarum* is often used in evolutionary ecology studies such as behavioural ecology^9^, host-parasite relationships^10,51^ or at the crossroads between parasitology and eco-toxicology^45,52,53^.

Already two decades ago, three cryptic lineages (A, B and C) were identified in German populations of *G. fossarum*^54^, and two of them were shown to have different habitat requirements^13^ and, likely, sensitivity to pollutants^20,21^. In recent years, the level of CD was further explored in several parts of Europe and has dramatically increased, establishing *G. fossarum* as a complex of highly divergent lineages that may represent cryptic species^55–59^. These studies revealed a substantial amount of CD occurring even at small geographical scales. Intriguingly, the number of lineages seems to be the highest in south-eastern Europe, but the highest phylogenetic diversity recorded so far is in central Europe^59^. However, the extent of diversity in *G. fossarum* is currently inadequately known and most likely underestimated at the continental level, because of numerous and wide sampling gaps. Indeed, most of the previous studies were restricted geographically compared to the known distribution of *G. fossarum* in Europe.

Thus, the present study is based on a large number of individuals (almost 5000) and a geographically extensive sampling scheme (ca. 500 sites), including both original and literature data, covering the morphospecies' geographic range in Europe. The mitochondrial DNA cytochrome C oxidase subunit I (COI) gene barcode region sequence^60^ has been chosen to assess the extent of CD. The reasons for that were: (1) sequences of this marker were widely available for *G. fossarum* in the literature and (2) this marker was successfully used at identifying CD both in *G. fossarum*^55–59^ and in other gammarids (e.g.^28^). Following Kekkonen and Hebert^61^, we used COI as a quantifier of diversity and an "efficient start for taxonomic workflow", only targeting here the delineation of Molecular Operational Taxonomic Units (MOTUs) as a way to propose testable "species hypotheses" (e.g.^17^).

Specifically, we aimed to:

1. estimate the level of hidden diversity within *G. fossarum* at the continental scale using MOTUs delimited with distance and tree-based methods,
2. reveal and interpret the critical temporal and geographic patterns of the observed diversification allowed by the pan-European sampling,
3. discuss HCD in relation to evolutionary, ecotoxicology, biodiversity surveys and biomonitoring as well as to the future of HCD assessment.

## Results

### A global overview of MOTU diversity

**Figure 2:**
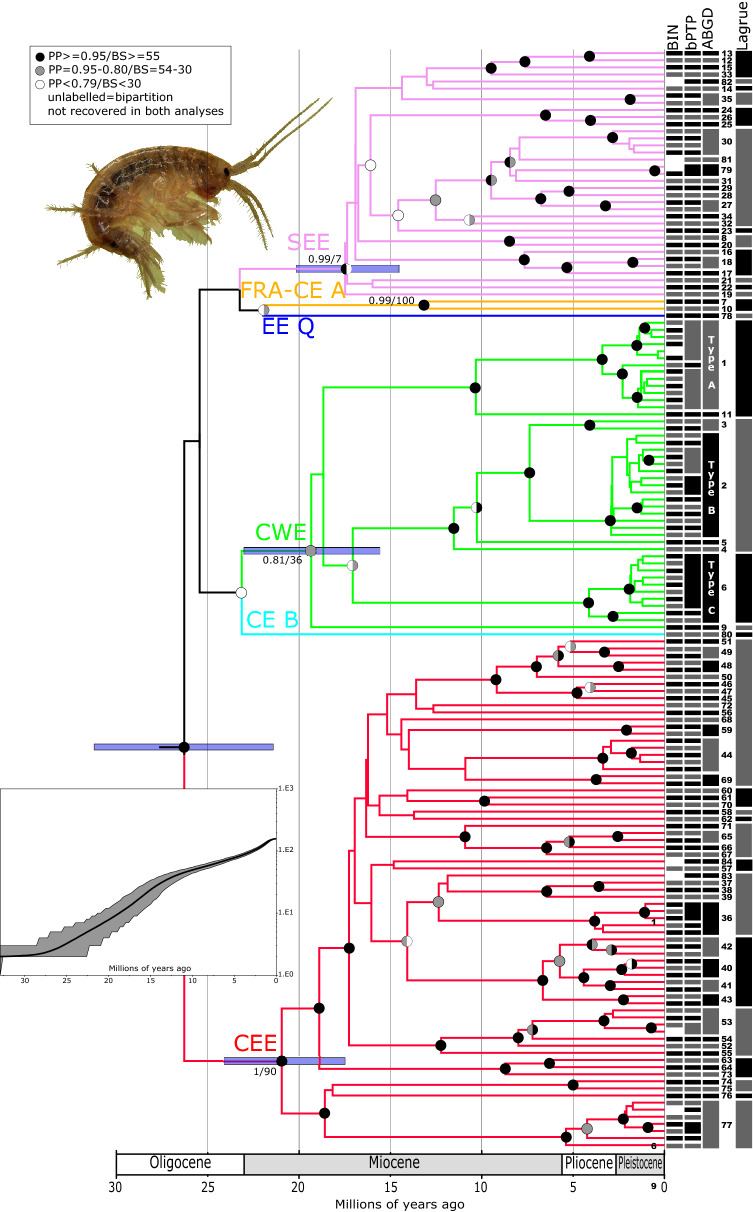
Bayesian maximum clade credibility chronogram with MOTU designation for *G. fossarum*. The outgroup is removed for clarity. Each BIN is represented by one randomly chosen haplotype. Grey bars at key nodes represent 95%HPD (Highest Posterior Density) intervals of clade age. Circles at nodes represent Posterior Probabilities (PP) for Bayesian Inference (BI) and as bootstrap values (BS) for Maximum Likelihood (ML, see Fig. S1), according to the following colour code: black: PP ≥ 95%/BS ≥ 55%, grey: PP = 95–80%/BS = 54–30% and white: PP < 79%/BS < 30%, unlabeled = bipartition not recovered in both analyses. Precise values are given for key nodes. Bars annotated on the right represent results of the MOTU delimitation methods i.e. Barcode Index Number (BIN), the Bayesian implementation of the Poisson Tree Processes (bPTP), the Automatic Barcode Gap Discovery (ABGD) and Lagrue's reproductive isolation methods, respectively. Three ABGD-MOTUs, 1, 2 and 6, corresponding to types A, B and C as defined by Müller et al. (2000), respectively are specified. Six major clades were distinguished and highlighted by the following colours: SEE (South-Eastern Europe, purple), FRA-CE A (France and Central-European A, yellow), EE Q (Eastern-Europe Q, dark blue), CWE (Central-Western Europe), CE A (Central-EuropeanA, yellow) and CEE (Central-Eastern Europe). First inset: Image of a male *G. fossarum *sensu stricto, i.e. Type A which occurs at the type locality (Photograph: Denis Copilaş-Ciocianu). Second inset: Lineages through time (LTT) plot. Analysis was performed on the dataset reduced to BINs. Grey lines represent 95%HPD. Tree was generated using BEAST 2.4.8 (https://www.beast2.org/).

**Figure 3:**
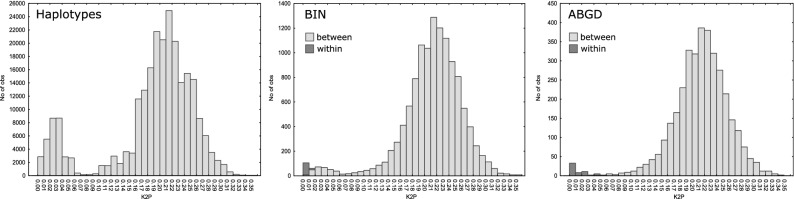
Kimura 2 parameter (K2P) distances (**a**) between haplotypes, (**b**) within and between BIN-MOTUs and (**c**) within and between ABGD-MOTUs.

COI sequences for the DNA-barcode region were either newly generated or derived from the literature for 4926 *Gammarus fossarum* individuals, from 498 sampling sites distributed throughout 19 European countries (Fig. 1), totalling 691 haplotypes (Table S1, Fig S1). An extraordinarily high level of cryptic diversity was evidenced by a total of 146, 152 and 84 MOTUs obtained with BIN, bPTP and ABGD methods, respectively (Fig. 2). TheK2P distance between haplotypes ranged from almost zero up to 0.35 (Fig. 3a). Although the distribution of genetic distance values was generally bimodal, only a small proportion of values centred around 0.03, while most concentrated around 0.22, illustrating that most haplotypes are highly divergent (Fig. 3a). Only 35 of the 146 BINs have already been known from literature or derived from the sequences mined from GenBank by BOLD, resulting in 111 new BINs in the present study, a fourfold increase. In addition, eight short sequences (397–468 bp) derived from the literature, divergent enough from the existing BINs not to be ascribed to any of them, but too short to be attributed their own BINs (following BOLD standard), should be considered as eight extra BIN-level MOTUs, extending the total to 154 BIN-equivalent MOTUs. Both BIN- and ABGD-MOTUs, shared a bell-shaped distribution ofK2P distances centred on 0.22, illustrating that most MOTUs are highly divergent (Fig. 3b,c). However, while the lowestK2P value among ABGD-MOTUs was 0.05, this value for BIN-MOTUs was occasionally down to < 0.01.

In 62 instances, MOTUs based on BIN, bPTP and ABGD methods were concordant. Single ABGD-MOTUs comprised from two to up to fifteen BINs, clearly showing that BIN was the least conservative among the applied MOTU delimitation methods (Fig. 2).

We observed 32 lineages being both sister clades in the phylogeny (Fig. 2) and diverging by > 0.2 K2P distance. These 32 Lagrue-MOTUs are a conservative estimate of the number of lineages for which we could expect pre-zygotic reproductive isolation as suggested by Lagrue et al.^57^.

### Spatial and temporal pattern of diversification

The Bayesian maximum clade credibility chronogram (Fig. 2) based on BINs, each represented by one randomly chosen haplotype, revealed the phylogenetic relationships and divergence times between MOTUs of the *G. fossarum* morphospecies (Fig. 2). Three significant points are emphasized. First, the estimated age of the entire lineage complex is ca. 26 Mya, i.e. middle-Oligocene. Second, all the major-clades appeared already in the early-middle Miocene, ca. 21–13 Mya. These two features illustrate the ancient age of the *G. fossarum* complex and its early diversification. Third, the Lineage Through Time analysis (LTT, inset Fig. 2) indicated a rather continuous evolutionary diversification, although at a somewhat slower pace from ca. 12.5 Mya.

**Figure 4:**
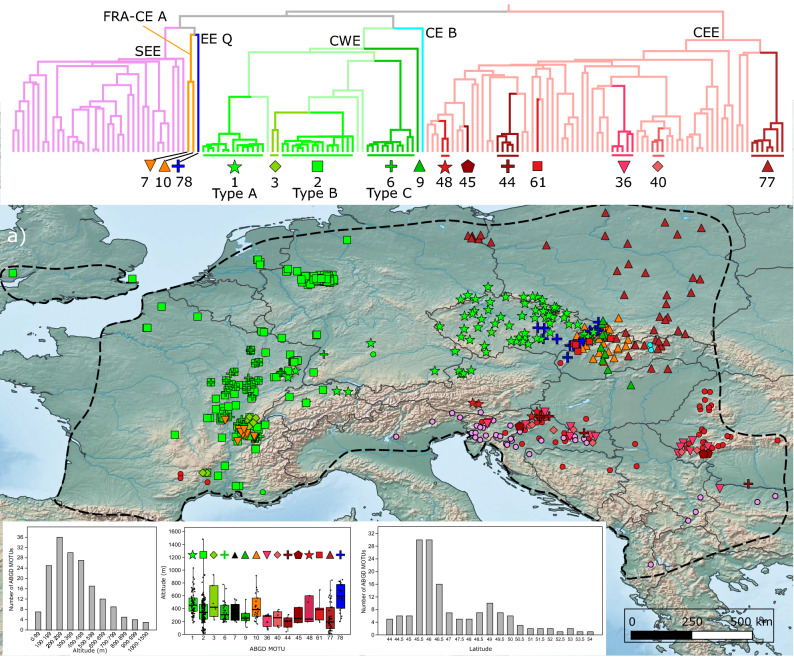
Geographical and altitudinal distribution of major clades and AGBD-MOTUs in reference to their phylogenetic position. (**a**) Thin blue lines on map present first-order river, bold grey lines are country borders. Dashed black line represents a simplified distribution range limits for *G. fossarum*. (**b**) Simplified version of the Chronogram from Fig. 2. The six major clades distinguished in Fig. 2 are highlighted by the same colour code, both on the map and on top inset tree: SEE (South-Eastern Europe, purple), FRA CE A (France Central-Europe A, yellow), EE Q (Eastern-Europe Q, dark blue), CWE (Central-Western Europe, green), CE B (Central-Europe-B, turquoise blue) and CEE (Central-Eastern Europe, red). The 84 ABGD-MOTUs were split into two categories: MOTUs with narrow distribution based on our sampling (< 100 km between the most distant sites) and more broadly distributed MOTUs. The colour code for major clades was followed. Presence of the narrowly distributed MOTUs is indicated with a circle on the map, while the more broadly distributed MOTUs were attributed a specific symbol (see the top inset tree for details). To prevent overlap of symbols in sites with co-occurrence, some positions are slightly shifted. (**c**) The histogram presents the absolute frequency of ABGD-MOTUs according to altitude. (**d**) The altitudinal distribution of broadly distributed ABGD-MOTUs based on ABGD method is presented as box plot. The colour code follows major clade designation of the top inset tree (see Fig. 2 for details). The dots represent sampling sites, thick black bar represent the mean, grey box represents first and last quartile, thin vertical line represents min–max values. (**e**) Histogram representing the number of lineages per latitudinal band. Maps created with QGIS 3.4.5 (https://www.qgis.org/fr/site/) and trees with BEAST 2.4.8 (https://www.beast2.org/).

Six highly divergent major clades (Fig. 2) SEE, FRA-CE A, EE Q, CWE, CE B and CEE (see below for explanation of acronyms) were recovered according to the rationale presented in Material and Methods , matching the patterns observed in previous multilocus studies. All major clades diverged anciently, around 20 Mya (Figs. 2, 4b). In the following interpretation, a clade was considered as (1) "narrowly distributed" when it was present at only one sampling site or several geographically close sites (< 100 km apart), (2) "broadly distributed" when sites harbouring this clade were > 100 km and < 1000 km apart, and (3) "widespread" when locations were spanning > 1000 km. The same rule was applied to BIN- and ABGD-MOTUs.

Among these six clades, three clades, i.e. CWE (Central-Western Europe), CEE (Central-Eastern Europe) and SEE (South-Eastern Europe), are widespread and rich in BIN- and ABGD-MOTUs. However, the MOTUs harboured by these clades can drastically differ in geographic range size, ranging from narrowly endemic to widespread (Fig. 4a). The CWE clade included 44 BIN- or BIN-equivalent MOTUs and eight ABGD-MOTUs. The clade itself is widespread, ranging from the UK and France in Western Europe, to south-western Poland and western Slovakia in central Europe (Fig. 4). Four ABGD-MOTUs within this clade are broadly distributed. Among them, ABGD-MOTU 3 is restricted to France but split between north-western Alps and south-eastern Massif Central, two areas > 500 km away from each other, each harbouring a specific BIN (Table S1). The ABGD-MOTU 9 is present in Slovakia, Hungary and Poland. The three other ABGD-MOTUs 1, 2 and 6 are more widespread. They would (see “Discussion”) correspond to the *G. fossarum* types A, B and C, respectively, described by Müller (2000)^54^ (Fig. 2) and is presented in detail below. Only three BINs in this CWE clade were presumably narrowly distributed (Fig. 4a,b, Table S1).

The CEE clade encompassed the highest number of MOTUs: 72 BIN-, or BIN-equivalent MOTUs and 48 ABGD-MOTUs (Fig. 2). Like CWE, the CEE clade is widespread although with a peculiar, discontinuous distribution. While it is present almost exclusively in Central and Eastern Europe, two BINs are nevertheless found in the southern Massif Central in France. The CEE clade includes seven ABGD-MOTUs that are broadly distributed to a various extent (Fig. 4a,b). The ABGD-MOTUs 48 and 61 were present in a few sampling sites ca 250 km away from each other. The ABGD-MOTUs 36, 40, 44 and 45 were present on each side of the Pannonian Basin, (in the south-eastern Alps and the Southern Carpathians), at least ca 500 km apart. The last ABGD-MOTU, 77 is the most widely distributed, being present in Central Europe, namely in Hungary, Slovakia, Poland and eastern Germany. The remaining 48 BINs are presumably endemic to eastern and south-eastern Europe (Fig. 4, Table S1).

The SEE clade harbours 35 BIN- or BIN-equivalent MOTUs and 28 ABGD-MOTUs (Fig. 2). This clade is present in seven countries, from northern Italy in the west, to Albania and Bulgaria in the southeast of Europe. Contrary to CWE and CEE clades, none of these MOTUs was either widespread or even broadly distributed to any extent (Fig. 4a,b).

The major clade FRA-CE A (France-Central-Europe-A) was represented by two ABGD-MOTUs, each being also a single BIN-MOTU (Fig. 2). These two MOTUs are located in two distinct geographical regions, distant by > 1000 km: one in the northern French Alps, the other in western Slovakia and southern Poland (Western Carpathians) (Fig. 4a,b).

The two remaining major clades EE Q (Eastern-Europe-Q) and CE B (Central-Europe-B) were represented only by a single ABGD-MOTU, 78 and 80, respectively, each being also a single BIN-MOTU (Fig. 2). The CE B clade is present only in the Western Carpathians, while the EE Q clade is slightly more broadly distributed in eastern Czech-Republic and westernmost Slovakia (Fig. 4a,b).

### ABGD-MOTUs latitudinal and altitudinal distribution

The diversity of ABGD-MOTUs also varied according to altitude (Fig. 4c). Although numerous MOTUs inhabit the zone between 100 and 500 m asl, some ABGD-MOTUs were found below 100 m and many above 600 m, even up to 1500 m. Among the 15 ABGD-MOTUs found to be broadly distributed (Fig. 4d), most had a rather wide altitudinal span. For example, ABGD-MOTU 2 occurs from the sea level to as high as 1500 m asl, while MOTU 78 was found in sub-mountain areas and some ABGD-MOTUs (e.g. 36, 40, 44 and 77) are mainly restricted to lowlands (Fig. 4d).

The diversity of ABGD-MOTUs also varied according to latitude (Fig. 4e), with two peaks – the most prominent from 45.5°N to 46.5°N and another, much lower, from 48.5°N to 50°N. The first one is associated predominantly with the diversity hotspots in the southern part of *G. fossarum* distribution range, namely the western and south-eastern outskirts of the Alps, the south-western part of the Pannonian Basin and the southern part of the Western Carpathians. The other peak illustrates the lineage diversity present in the Western Carpathians predominantly. The lowest lineage diversity is observed between 50.5°N and 54.0°N, corresponding mainly to the western and central parts of the Great European Plain.

### Historical types A, B and C

**Figure 5:**
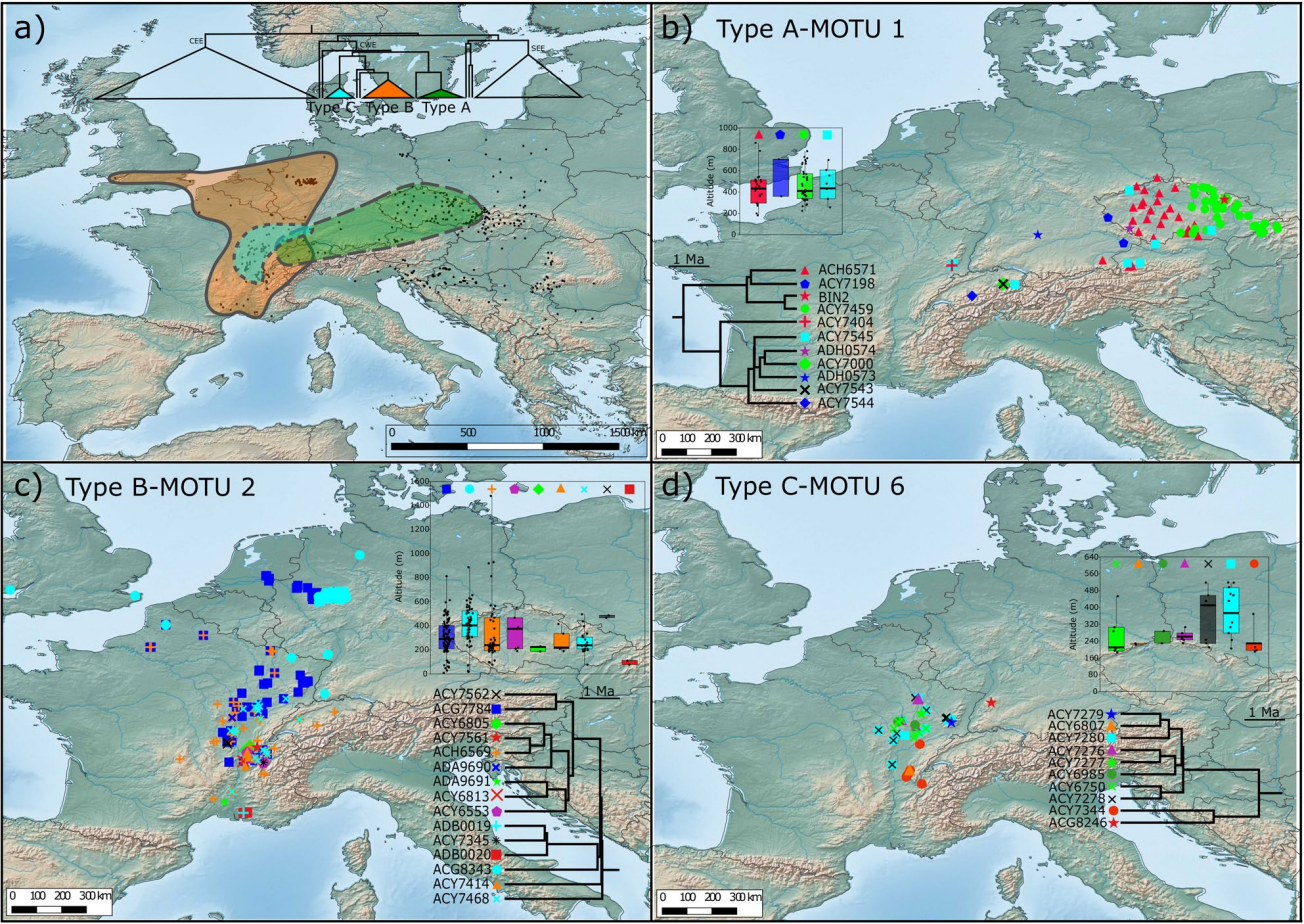
Geographical and altitudinal distribution of the three AGBD-MOTUs 1, 2 and 6, corresponding to types A, B and C as defined by Müller et al. (2000), respectively, and the contained BIN-MOTUs. (**a**) General distributional range of the three ABGD-MOTUs, 1, 2 and 6 , corresponding to type A, B and C as defined by Müller et al.^54^ (2000), respectively. Thin dashed line indicates the distribution of each ABGD-MOTU. The tree presented is a collapsed version of the chronogram highlighting the phylogenetic position of key MOTUs. (**b**–**d**) Distributional maps of BIN-MOTUs within each the three ABGD-MOTUs 1, 2 and 6, respectively. To prevent overlap of symbols in sites with co-occurrence, some positions are slightly shifted. Each presents both, the phylogenetic relationship between BIN-MOTUs and the symbols used in the map and in the box plot. Box plots show the altitudinal distribution of each BIN-MOTU. Dots represent sampling sites, thick black bar represent the mean, grey box represents first and last quartile, thin vertical line represents min–max values. Maps created with QGIS 3.4.5 (https://www.qgis.org/fr/site/) and Trees with BEAST 2.4.8 (https://www.beast2.org/).

As shown on Figs. 2 and 4, the ABGD-MOTUs 1, 2 and 6 would correspond, respectively, to the *G. fossarum* types A, B and C initially defined by Müller^54^ (see “Discussion” for details). Since these MOTUs were the most commonly used for various ecological and ecotoxicological studies (e.g.^20,21^), it is important to highlight their biogeographical history. They include large numbers of BINs, 12, 15 and 10, respectively (Figs. 2, 4, 5b–d for details) and their geographic ranges overlap either fully (B and C) or only partially (Fig. 5a). Numerous cases of sympatry between these types were also observed in France, especially in case of ABGD-MOTU 2 (type B) and 6 (type C), and ABGD-MOTU 2 (type B) and 6 (type C) (Fig. 5b–d). Altitudinal ranges for each BIN-MOTUs present in at least three sampled sites are shown in box-plot insets in Fig. 5b–d. The ranges observed at the ABGD-MOTU levels are overall as wide at the BIN-MOTU level for ABGD-MOTUs with high sampling effort. It shows that the ranges of ABGD-MOTUs were not artefacts resulting from combining BIN-MOTU ranges which would have non-overlapping distributions.

### Geographic overlap and co-occurrence of BIN-, ABGD-MOTUs and major clades

We found a regional co-occurrence of BIN-MOTUs, ABGD-MOTUs and major clades (Figs. 4, 5). To further quantify within-site co-occurrence, sites with two or more sampled individuals (321 out of 498 sites) were used. Within-site co-occurrence of at least two BIN-MOTUs, ABGD-MOTUs and highly divergent clades was observed in 32.4%, 17.8% and 7.2% of the sites, respectively. Also, at least three BIN-MOTUs co-occurred in 19 locations and at least three ABGD-MOTUs in four sites (Table S1). Sympatry between these different genotypes is therefore quite frequent. Sampling size is likely to impact the ability to detect co-occurrence, particularly if MOTUs are unevenly distributed, which seems a regular feature for *G. fossarum* (e.g.^55^). For example, in our dataset, rare BIN-MOTU had a frequency of no more than 0.2 in 73.6% of the sites with more than ten individuals sampled, and no more than 0.1 in 49.1% of such sites.

### Geographic hotspots of divergence

**Figure 6:**
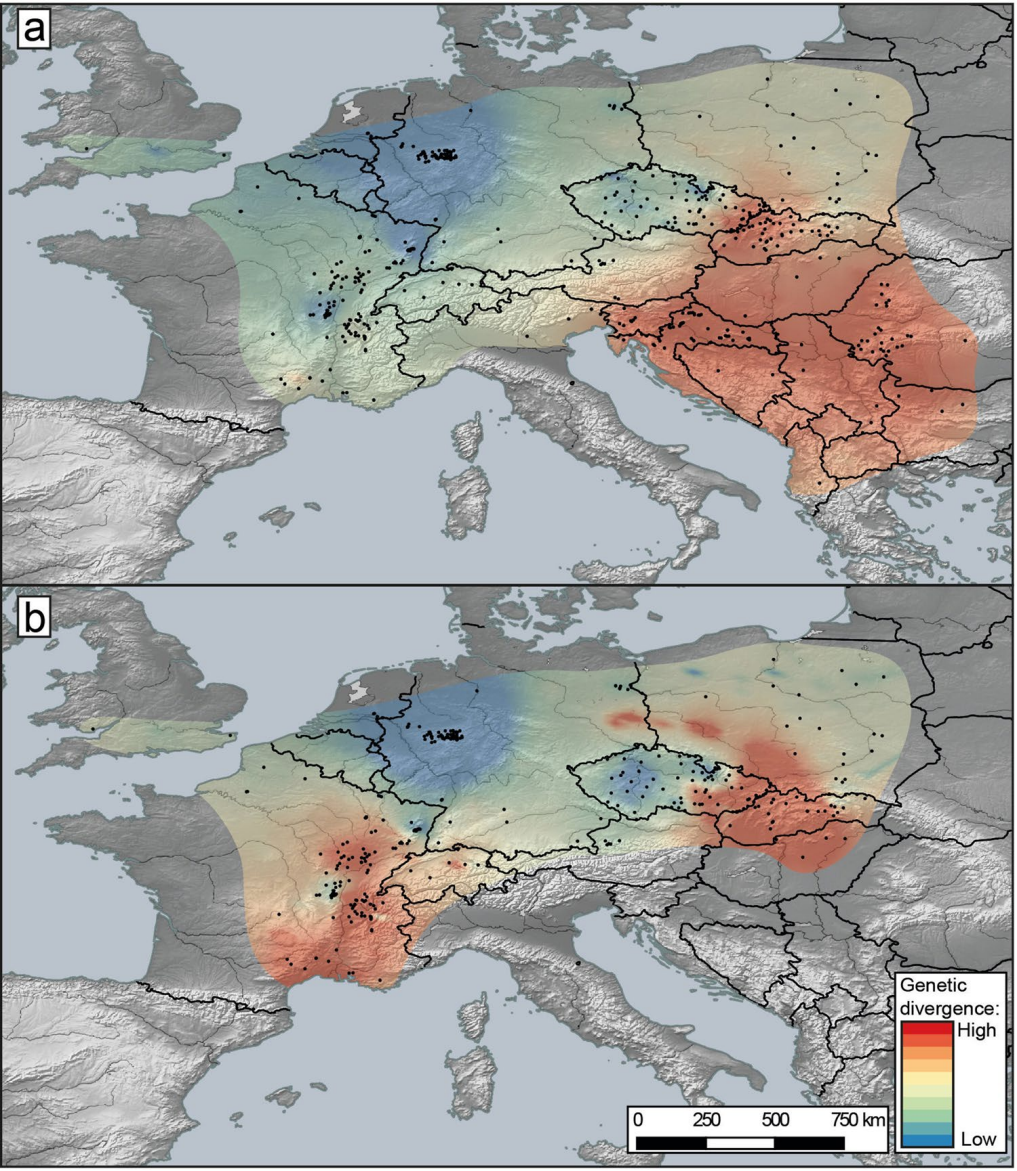
A genetic landscape map based on COI mtDNA haplotypes overlaid onto a relief map of Europe. Black dots indicate sampling sites. Warmer colour (red) reflects high molecular divergence between neighbouring localities, while colder colour (blue) corresponds to areas of lower molecular divergence. (**a**) Map including all the sampling sites (498) and all haplotypes (691). (**b**) Map excluding highly divergent lineages endemic to the Carpathian, Dinaric and Pannonian regions (hotspots of most ancient divergence), to better illustrate the level of genetic divergence in the remaining area (469 haplotypes and 336 sites). Genetic distances were generated with Alleles in Space (AIS; https://www.marksgeneticsoftware.net) and maps created with QGIS 3.4.5 (https://www.qgis.org/fr/site/).

The landscape genetic map (Fig. 6a) of molecular divergence showed that the Carpathian, Dinaric and Pannonian regions exhibit the highest levels of lineage divergence. This pattern holds regardless if one or more of the six major clades are present in a given region.

If highly divergent endemic Carpathian, Dinaric and Pannonian lineages are excluded (i.e. explaining most of the previously described hotspots), the Western Carpathians still appear as a hotspot of high divergence with five major clades being sympatric in this area: CEE (ABGD-MOTU 77), CWE, EE Q, FRA-CE A and CE B (Fig. 6b). Many regions in France also appeared as local hotspots either, associated with the sympatric occurrence of two major clades, e.g. north-western Alps (CWE and FRA-CE A) and southern Massif Central (CWE and CEE) or divergent ABGD-MOTUs from the same major clade (e.g. 1, 2 and 6, i.e. type A, B and C in central-eastern France) (Fig. 6b). On the contrary, the Great European Plain (northern France, Germany, Poland) of the current distribution of *G. fossarum* appeared as an area of low divergence (Fig. 6a,b).

## Discussion

Our results, based on extensive continental scale sampling of *G. fossarum*, revealed a remarkable level of hyper cryptic diversity (HCD) with 146, 152 and 84 MOTUs obtained with BIN, bPTP and ABGD methods, respectively. The age of the species complex was estimated at ca. 26 Mya and the divergence was predominantly high, most lineages exhibiting a K2P distance of 0.22. Finally, a relatively high level of sympatry was observed even at the site level. Although CD was already documented in *G. fossarum*^54–59^, including HCD^58^, the present study considerably increases its extent, offering for the first time the opportunity to discuss its origin on a continental scale.

CD is a common phenomenon in freshwater morphospecies of the genus *Gammarus*, being reported in all Eurasian wide-ranged taxa studied thus far: e.g. *G. balcanicus*^5,33,62^, *G. komareki*^32^, *G. ochridensis*^29^, *G. pulex*^57,63,64^, *G. roeselii*^28^, *G. lacustris*^32^, *G. kischineffensis*^64^*, G. leopoliensis*^65^, but also in North American (e.g. *G. pecos*^37^) or East Asian (e.g. *G. nekkensis*^66^) taxa. HCD was detected in three studies^5,28,32^. Two studies identified 15 and 35 ABGD MOTUs in *G. roeselii*^26^, and in *G. komareki*^30^, respectively. A survey covering the entire European range of *G. balcanicus*^5^ presented 49 highly divergent lineages (likely to represent ABGD-MOTUs given their degree of divergence). In comparison, a previous study^62^ on the same taxon reported up to 22 lineages in the Carpathian Mountains alone. These studies shared a high sampling effort, both in the number of sampling sites and individuals, pointing out the need for appropriate sampling strategies to detect HCD. The age of these species complexes was usually shown to be old, ranging from 4–6 Mya in *G. kischineffensis*^64^ or *G. leopoliensis*^65^ to 15–17 Mya for *G. balcanicus*^5^ and *G. roeselii*^26^. Intriguingly, the high frequency of sympatry observed between *G. fossarum* MOTUs was not detected in the other species complexes^5,32^.

Cryptic diversity is common in crustaceans, even overrepresented when compared to other metazoans^2^. The CD in amphipods is far from being restricted to the genus *Gammarus*. It has been reported for many other epigean freshwater amphipod families, e.g. North American Hyalellidae^30,38,39,67^, New Zealand Paracalliopiidae^31^ or Australian Chiltonidae^34^. It is also extensively known from hypogean freshwater taxa, e.g. Niphargidae^68,69^, Paramelitidae^70^, or Crangonyctidae^71^, as well as in marine amphipods, including abyssal habitats, e.g. Epimeriidae^72^, Eusiridae^73^, Eurytheneidae^74,75^. Among all these families, examples of both high/old divergence^69,70,75^ and HCD^34,38,70^ are not rare. Therefore amphipods, whatever the habitat, are highly prone to CD. The present, often decades old, morphological definitions of amphipod species seem insufficiently detailed in many cases. Either morphological stasis, convergent or parallel evolution could be reasons explaining this pattern, but discussing evolutionary processes in detail is beyond the scope of the present paper. It might also be a matter of taxonomic impediment, associated with an increasing shortage of taxonomists^76^, amphipods ranking amongst the least taxonomically studied taxa^77^.

Our data reveal an old and highly complex pattern of molecular diversity within a morphospecies at the local and the continental scale. For example, the ancestors of three clades likely had a pan-European distribution. First, while the CEE clade geographic distribution is centred in central and eastern Europe, three ABGD-MOTUs 68, 71 and 72 were located in southern France, suggesting that the ancestor of this clade had a pan-European distribution. Additional supporting evidence comes from AGBD-MOTU 71, which is in a well-supported sister relationship with the ABGD-MOTUs 65–67, all the latter currently restricted to Romania. The estimated age of this CEE group of lineages is ca 11 Mya, i.e. middle Miocene. Second and similarly, the two MOTUs of the clade FRA-CE A, one restricted to France, and the other to Czechia and Slovakia, are well-supported sister MOTUs, which diverged in Mid-Miocene, ca 12.5 Mya. Third, MOTUs of the CWE clade were present in Western Europe (ABGD-MOTUs 2, 3, 6) and Central Europe (ABGD-MOTUs 1, 9), and also diverged during the Miocene.

Local presence of high CD has already been known for several, predominantly submontane regions of southern and central Europe^58^. We show here a general trend that confirms this pattern and provides further evidence for other local diversity hotspots in the south-western Pannonian Basin and the south-western outskirt of the Alps. However, we point out that the Balkan region is still poorly covered and likely harbours a significant amount of diversity. The probable middle Oligocene (ca. 26 Mya) to late Pliocene (ca. 3 Mya) timeframe of diversification for the main clades and MOTUs corresponds well with the temporal scheme obtained for other widespread European freshwater amphipods, such as *G. balcanicus*^5^ or *G. roeselii*^28^. Our results advocate Alpide orogeny, i.e. simultaneous uplift of the Alps, Carpathians, and Dinarides, as the main factor shaping the phylogeography of *G. fossarum*. This period was also associated with the presence of extensive lacustrine systems and marshlands covering vast areas of the continent, subsequently fragmented as a result of the regional uplifts^78^. This could enhance the spread of some lineages over vast areas, promoting the subsequent diversification process. That is the case of the sister ABGD-MOTUs 7 (FRA) and 10 (CE A), the former being endemic to the western outskirts of Alps and the latter to the Western Carpathians, while both are in a sister relationship to another highly divergent ABGD-MOTU from the Western Carpathians. We also reveal the presence of wide areas of reduced genetic diversity, predominantly on the Great European Plain, that was partly covered by glaciers during the Last Glacial Maximum (LGM) and partly by a permafrost tundra with few scattered patches of forest^79^. Thus they provided no favourable conditions for most of the *Gammarus* species, which are known to play a major role in decomposing broadleaved tree debris^41,80^. In consequence, the area was probably colonised only after the LGM, as is evidenced already for two largely sympatric BINs of the ABGD-MOTU 77 that spread over the central part of the Great European Plain from the glacial refugium/diversity hotspot in the Western Carpathians^81^. Similarly, the other widespread ABGD-MOTU 2 is represented by three BINs in the western part of the Great European Plain. Overall, our findings stress the fundamental role played by a combination of regional historical and environmental factors shaping the local genetic diversity of the *G. fossarum* complex in different parts of Europe and point out to the need for precise identification of the sampled populations with DNA barcodes.

The *G. fossarum* types A, B and C were defined in by Müller^54^ in 2000 based on 16S rRNA sequences and allozyme data from samples collected mainly in Germany. More recent publications referring to these three types were based on COI, but the sampling remained restricted to or centred around Germany^20,55,56,63^. Our study shows that samples from these studies^20,55,56,63^ are associated exclusively with one or two BINs per type (type A: ADH0573^20^; type B: ACG7784 and ACG8343^20,52,53^; type C: ACG8246^53^). We conclude that our ABGD-MOTUs 1, 2 and 6 correspond to types A, B and C, respectively, each type comprising many BIN level MOTUs (11, 15 and 10, respectively). Our study confirms that the geographic distribution of type A does not extend westward more than already reported in the literature^54,58,82^. However, our results substantially extend the distribution of type B (but see^55^) and even more notably type C. Type B was known to be widely present in Germany, Switzerland and north-eastern France^54,57,63^ and was recently recorded in the UK^40^. Our study extends its distribution to north-western and southern France. Initial studies restricted the distribution of type C to the French-German border marked by the Rhine River^20,54,63,82^. The present study shows it is widely present also in the whole eastern part of France. Some studies pointed out that geography could alone be predictive of the distribution of ABGD level MOTUs, e.g. type A and B within Germany^54^. However, the co-occurrence of individuals associated with different types was also pointed out in the literature (e.g.^13^). Based on our substantial sampling effort and extended geographic cover, we conclude that co-occurrence of MOTUs (ABGDs, BINs) may be the rule for the distributional pattern of *G. fossarum*. Initially proposed to be of pre-Pleistocene origin^54,82^, the evolutionary splits between types A, B and C are in our study suggested at early Miocene, matching the early diversification of the CWE clade itself. Phylogeographic history of type A was already extensively studied^54^. Detailed analysis of other types is beyond the scope of the present paper. However, intricate diversification patterns including multiple refugia and more or less extensive expansion during each interglacial in the Pleistocene and part of the Pliocene are likely, as was already proposed for two stygobiotic amphipods (*Niphargus virei*^83^, *N. rhenorhodanensis*^84^) in the same area.

It is essential that HCD is taken into account in both basic and applied studies while ignoring it might have multiple potential impacts. Recently, accumulating studies upon *G. fossarum* show that CD may impact various types of biological processes, including (1) sexual selection with impact on the outcome of mate choice and male-male competition^9^, (2) intensity of infection by acanthocephalan parasites^10,11^ and parasite-induced mortality^10^, (3) functional ecology associated with habitat partitioning^13^ and (4) ecotoxicology, with possibly different response to a contaminant^20,21^. It might also be largely involved in the within-species biodiversity effects, increasingly acknowledged in the biodiversity-functioning relationships^85^. Generally, how many different Evolutionary Significant Units (ESU) should be considered within *G. fossarum* is a relevant question and a challenge as exemplified by other amphipods^35,86^. On the one hand, our results suggest that individuals of different types will barely interbreed as they belong to different Lagrue-MOTUs. Indeed, it was revealed that individuals from BINs diverging by ca 16% K2P distance (ACY7276, type C vs either ACY7784 or ACH6569, both type B) predominantly discriminate between each other^9,57^. On the other hand, the same authors also found out that individuals from the two studied BINs within type B (ACY7784 or ACH6569) diverging by ca. 4% K2P distance, mate randomly, exhibiting no apparent pre-zygotic reproductive barriers. Our study illustrates that many BINs within each type are sympatric, so the question of how frequent between-MOTU mating remains open but testable. Applying nuclear markers, such as microsatellites (which are available for *G. fossarum*), might help to answer this question in the future^14^.

Our study points out a tremendous haplotypic (691 haplotypes) and MOTU-wise diversity as well as to the co-occurrence of MOTUs at the same sampling site. Also, such syntopy often includes rare MOTUs. Both phenomena will impact the future exploration of CD in *G. fossarum*, either for basic (e.g. phylogeography) or applied (e.g*.* monitoring or ecotoxicology) topics. Undoubtedly, the sampling strategy should include enough individuals per site to detect rare MOTUs. The Sanger sequencing of single PCR amplicons^87^, targeting the full COI barcode (as in the present study), might convey highly informative content but, simultaneously, it is a costly low throughput method^87^. Pyrosequencing of mitochondrial 16S targeting diagnostic Single Nucleotide Polymorphisms (SNPs) has been applied to identify type A, B and C^82^. Being high-throughput and fully valid in the restricted geographic area where it was initially implemented (mainly Switzerland), the method would seem inappropriate if applied on a continental scale. More recently, e-DNA-metabarcoding was used to detect the presence of *G. fossarum* in UK rivers. Environmental DNA (eDNA) was amplified for the entire macroinvertebrate community^40^, and a 333 bp COI mini-barcode was sequenced using high throughput sequencing (HTS^88^). The method proved (1) to be sensitive at detecting *G. fossarum* even at very low abundance (2.6% of total biomass), and (2) discriminant, as the mini-barcode proved informative enough compared to the full barcode to allow unambiguous phylogenetic assignment at the ABGD-MOTUs level (i.e. type B). Would this mini barcode be sufficiently informative to discriminate all BIN-MOTUs and ABGD-MOTUs diversity at the European scale? Although being outside the scope of the present paper, the large amount of data associated with the current study would easily allow testing it in silico.

Given the HCD described for *G. fossarum* in the present paper, we recommend any research, either related to applied or basic topics, to obtain both COI barcodes and BINs for the individuals under study. Our research also points out the necessity of a comprehensive DNA barcode reference library^89^ for *G. fossarum* at the European scale. Even if our research is based on an extensive sampling effort, such sampling is heterogeneous, and many areas of *G. fossarum* distribution are unsampled or undersampled. Even if the task will be tremendous, particularly given the shortage of morpho-taxonomists, defining and naming the MOTUs would be desirable^90^ as recently occurred for ABGD-MOTU 77, which was ascribed a formal taxonomic name: *Gammarus jazdzewskii*^81^. In the same line, as *G. fossarum* offers the opportunity to relatively easily set up crossing experiments^57^, a feature rarely explored in amphipods in the context of CD, it should be further explored^37^.

## Methods

### Data overview

Sequences for COI were derived from a total of 4926 individuals collected from 498 sites (Fig. 1, Table S1). The sampling included 19 countries, covering the whole range of *G. fossarum* in continental Europe as well as two sites from United Kingdom, an area considered as recently invaded^40^. The collected data included: (1) original data (206 sites, 2071 sequences), (2) data derived from the co-authors' previously published studies^57–59,91^ (198 sites, 682 sequences, although 463 sequences from^54^ were initially unreleased in GenBank) and (3) data derived from other published papers^20,40,55,56,63,92,93^ (94 sites and 2173 sequences, although 2086 of these sequences^56^ originated from a very restricted area in Germany). All sequences retrieved from GenBank were checked to be phylogenetically part the *Gammarus fossarum* species complex using both BLAST (https://blast.ncbi.nlm.nih.gov/Blast.cgi^94^) and assessing monophyly relative to sister clades^93^. Sampling size per site was on average 10 individuals but was highly variable with a standard error of 14. A total of 180 sites contained only one individual, 95% originating from already published studies (Table S1). Sampling effort was variable (Fig. 1). Some countries e.g. Slovakia and The Czech Republic were evenly and highly sampled but with low number of samples per site while other countries e.g. Germany accounted for few sites but heavily sampled.

### Processing of newly gathered material (sampling, taxonomic identification, molecular methods)

The newly gathered material (Fig. 1) was collected using kick-sampling with a benthic hand-net and fixed in 96% ethanol on site. In the laboratory, the animals were identified to the morphospecies level using characters described in available keys^95–99^. Genomic DNA was extracted from pieces of muscle tissue using either standard phenol-chloroform^100^ or Chelex^101^ protocols. For all individuals the barcoding region of the mtDNA cytochrome C oxidase subunit I (COI) was amplified for 659–710 bp long fragment (including primers) using any of the following primer pairs: LCO1490 and HCO2198^102^, UCOIF and UCOIR^103^, COIGrF and COIGrR2^28^, LCO1490-JJ and HCO2198-JJ^104^. Details about primer sequence and location are given in Table S2. All details of the molecular procedures followed those described by Mamos et al.^5^. The amplicons were sequenced using the BigDye sequencing protocol (Applied Biosystems 3730xl) by Macrogen Inc., Korea. Sequences were edited and aligned with CLUSTALW 2.0 (https://www.clustal.org/)^105^ using either MEGA 7 (https://www.megasoftware.net/)^106^ or Geneious 6.0.5 (https://www.geneious.com/). Based on a 530 nucleotides long alignment, haplotypes were retrieved using DnaSP v5 (https://www.ub.edu/dnasp/)^107^. All newly produced sequences were checked to be phylogenetically part the *Gammarus fossarum* species complex using both BLAST (https://blast.ncbi.nlm.nih.gov/Blast.cgi)^94^) and assessing monophyly in relation to sister clades^93^.

### Cryptic diversity—MOTU delimitation

To explore the number of MOTUs that could represent putative cryptic species within *G. fossarum*, we applied four different approaches. Two methods were purely genetic distance-based methods, i.e. the Automatic Barcode Gap Discovery (ABGD)^108^ and the Barcode Index Number (BIN)^109^. A third one was a tree based phylogenetic approach, using the Bayesian implementation of the Poisson Tree Processes (bPTP)^110^. The fourth method takes advantage of the study of Lagrue et al.^57^ which combined genetic distance and reproductive isolation experiments. Both BIN and ABGD share the principle of clustering sequences into MOTUs according to their molecular divergence using algorithms aiming to find discontinuities, i.e. a so-called barcode gap separating intra- and interspecific genetic distances. For the ABGD (https://wwwabi.snv.jussieu.fr/public/abgd/) method, we used primary partitions as a basis for group definition. Primary partitions are typically stable over a wide range of prior values, minimise the number of false-positive (over-split species) and, in the absence of cryptic diversity, are usually found to reflect the number of taxa described by morpho-taxonomists^111^. The Kimura two-parameter (K2P) substitution model was applied^60^, and the default value of 0.001 was used as the minimum intraspecific distance. Given that neither morphology^32,55,103^ nor reproductive isolation^57^ provides any consensus about which maximum intraspecific distance allows for reliable species delimitation in amphipods, we explored a set of values of up to 0.1. The BIN algorithm is implemented as part of The Barcode of Life Data Systems (BOLD; https://www.boldsystems.org/)^112^. In this algorithm, the newly submitted COI sequences are aligned and compared pairwise and also to each sequence already deposited in BOLD. Then, the initial single-linkage clustering is performed on the aligned sequence data in such a way that each cluster is allowed for a maximum intra-cluster distance of 0.022, and its distance to any other cluster is more than twice the threshold (> 0.044). In the subsequent step, the clusters are refined in the sense that clusters whose members show high sequence variation, but lack discontinuity remain as a single MOTU, while those in which sequence variation shows clear internal partitions are assigned to two or more MOTUs, even if their separation is less than 0.022. Then, finally, each refined cluster is assigned a globally unique and specific identifier. For details on the BIN system see Ratnasingham and Hebert (2003)^109^. Like ABGD, in the absence of cryptic diversity, BINs are claimed to be congruent with the number of taxa described by morpho-taxonomists^109^. In addition, BINs may be registered and publicly available through BOLD, allowing tractability and have similar properties as classic taxonomy descriptors (e.g. can be split or synonymised). Only sequences over 500 bp can be included in the BIN clustering, while shorter sequences which are over 300 bp may be only ascribed to an existing BIN, but will not create a BIN or split an existing one. For example, our data set included eight short sequences derived from the literature, divergent enough from the existing BINs not to be ascribed to any of them but too short to be ascribed their own BIN (following the BOLD standard). In the result, we considered them as separate BIN-equivalent MOTUs.

The tree-based bPTP method uses non-ultrametric phylogenies. It incorporates the number of substitutions in the model of speciation and assumes that the probability that a substitution gives rise to a speciation event follows a Poisson distribution. The branch lengths of the input tree are assumed to be generated by two independent classes of Poisson events, one corresponding to speciation and the other to coalescence^110^. For the input tree, we used a Maximum likelihood (ML) tree obtained with RAxML HPC 8.2.9 (https://cme.h-its.org/exelixis/web/software/raxml/)^113^ from the haplotype data. The ML analysis was run under a thorough tree search with the GTR + G substitution model applied to each codon partition. The bPTP (https://species.h-its.org/ptp/) analysis was performed with 500,000 iterations of MCMC and 10% burn-in. Three runs were performed. The convergence of each run was verified through stationary pattern of the MCMC iterations trace plot. All runs provided congruent results.

The fourth method is newly introduced in the present paper and will be designated as the Lagrue-MOTUs. It takes advantage of the study of Lagrue et al.^57^, which combined COI K2Pdistance and reproductive isolation in *Gammarus fossarum*. Lagrue et al.^57^ tested two distance classes ca. 4% (for which reproductive isolation was absent) and ca 16% (for which reproductive isolation was almost exclusively present). Conservatively, sister clades that diverged by > 20% K2P distance in the present study were considered as different Lagrue-MOTUs and a surrogate of the minimum number of biological species.

### Time-calibrated phylogenetic tree

The time-calibrated phylogenetic tree was reconstructed using Bayesian inference (BI) in BEAST 2.4.8 (https://www.beast2.org/)^114^. A *Gammarus roeselii* sequence (KP789695) was used as an outgroup for the analysis. Two sets of data were used, one at the haplotype level the other at the BIN level. The outgroup was removed after the analysis. Priors for the substitution models were selected using bModelTest (https://github.com/BEAST2-Dev/bModelTest/)^115^. The best-fitting model of substitution in both cases was the transversion model (TVM) with gamma-distributed rate heterogeneity (G) and a given proportion of invariable sites (I). The log-normal relaxed clock with the Birth–Death speciation model was set as priors following the results of path sampling selection^116^. The recent cross-validated analyses of molecular clock, based on geological events for *G. fossarum*^58,59^ and other gammarids (e. g.^5,28,29^), indicated that the widely used arthropod COI rate of 0.0115 substitutions per site per My^117^ is useful in linking present-day patterns with historical processes. Thus, we have used it to calibrate the molecular clock in our phylogeny reconstruction. Four MCMC chains were run for 50 M iterations and sampled every 5000 iterations. The Effective Sampling Size (ESS) of each parameter was verified to be above 200 in Tracer 1.7 (https://beast.community/tracer)^118^. The runs were combined in LogCombiner 2.4.8, with 25% burn-in, and the maximum clade credibility chronogram was annotated using TreeAnnotator 2.4.8, both programs being part of BEAST 2.4.8 (https://www.beast2.org/)^114^.

In order to provide additional support for the BI topology, we have also reconstructed a phylogeny using the Maximum Likelihood approach (ML) in RAxML HPC 8.2.9 (https://cme.h-its.org/exelixis/web/software/raxml/)^113^. The best-scoring ML tree was searched under the GTR + G substitution model which was applied to each codon partition. Statistical support was estimated with the GTRCAT model of rate heterogeneity and 1,000 rapid bootstrap (RBS) replicates^119^.

The history of diversification was explored with the help of a Lineage Through Time (LTT) plot, generated with Tracer 1.7^118^, using 1,500 post burn-in trees that resulted from the BEAST analysis on the BIN dataset.

### Definition of major clades

We defined the major clades following studies from Copilaş-Ciocianu and Petrusek^58^ and Copilaş-Ciocianu et al.^59^ which were based on mitochondrial (COI and 16S) and nuclear markers (18S, 28S and EF1α). These studies have robustly recovered these deep clades in multilocus concatenation and species tree analyses. We acknowledge that, in the present study, node supports are not high in all cases as only the highly variable COI marker was used, which has reduced ability to resolve deep nodes (see “Results”). In addition, the names and acronyms were kept in order to maintain continuity with clade nomenclature from these previous studies^58,59^. Such clades refer to geography (i.e. SEE: south-eastern Europe), illustrating rather in which part of Europe a given clade has a centre of its distribution than willing to match any predefined strict geographic units. In addition, it is to be noted that extending the present study analysis at a continental scale might also have been associated with extending the original distribution of some clades.

### Geographic patterns of genetic diversity

The spatial diversity pattern of *G. fossarum* in Europe was illustrated with genetic landscape shapes generated with Alleles in Space (AIS; https://www.marksgeneticsoftware.net) software^120^. The genetic landscape visualizes the abrupt transitions between populations and groups of populations characterized by divergent haplotypes. The analysis followed the approach already applied to amphipods (e.g.^5^), i.e. converting the genetic distances between sites into a hypsometric map. First, using the AIS software, the genetic distances between sampling sites were calculated based on the COI sequences longer than 496 bp (in order to obtain a gap-free alignment) and connected into a network based on the Delaunay Triangulation. The genetic distance values were set in the midpoints of each connection in the network. The raw genetic distances acquired from the program were interpolated afterwards and the matrix of the 'elevation' values, with their respective latitude and longitude coordinates, was then imported into QGIS (https://qgis.osgeo.org) software to produce a genetic divergence surface image using the Triangulated Irregular Network (TIN) algorithm. The resulting image was plotted onto a relief map of Europe to create a final map in which the hypsometric tints reflect the genetic distance between population pairs. Two maps were constructed, one that included all the sampling sites (498) and all haplotypes (691) and another one which excluded the highly divergent lineages endemic to the Carpathian, Dinaric and Pannonian regions (the hotspots of the most ancient divergences, see “Results”), to better illustrate the level of genetic divergence in the remaining area. A total of 336 sites and 469 haplotypes were included in this latter analysis.

### Ethical approval

All the applicable international, national, and/or institutional guidelines for the care and use of animals were followed and were in agreement with recommendations from the "Comité d'Ethique et de l'Expérimentation Animale (C2EADijon Grand Campus) from Dijon University, France. No experiments on alive animals were performed for this study.

## Supplementary information

Supplementary Figure S1.

Supplementary Captions.

Supplementary Table S1.

Supplementary Table S2.

## Data Availability

All newly produced COI sequences are released in GenBank under accession numbers: MT978656–MT980726. The 463 sequences from Lagrue et al.^57^ which were initially unreleased in Genbank are now available under accession numbers: MT411018-MT411480. Sequences and metadata are available in BOLD dataset DS-GFOSCDEU (dx.doi.org/10.5883/DS-GFOSCDEU). All the new material used in this study has been stored in the permanent collection of either Biogeosciences Laboratory, University of Burgundy-Franche-Comté or the Department of Invertebrate Zoology and Hydrobiology, University of Lodz, Poland.
