# Supplementary material for: Continental-scale patterns of hyper-cryptic diversity within the freshwater model taxon Gammarus fossarum (Crustacea, Amphipoda)
Source: Sci Rep. 2020 Oct 6;10:16536. doi: 10.1038/s41598-020-73739-0 (PMC7538970; doi:10.1038/s41598-020-73739-0)
Supplement: Supplementary file 2 — Supplementary Captions. [file 41598_2020_73739_MOESM2_ESM.docx]

# Supplementary Material

**Continental-scale patterns of hyper-cryptic diversity within the freshwater model taxon *Gammarus fossarum* (Crustacea, Amphipoda)**

Remi Wattier, Tomasz Mamos, Denis Copilas-Ciocianu, Mišel Jelić, Anthony Ollivier, Arnaud Chaumot, Michael Danger, Vincent Felten, Christophe Piscart, Krešimir Žganec, Tomasz Rewicz, Anna Wysocka, Thierry Rigaud & Michał Grabowski

Fig-S1. Maximum-Likelihood (ML) tree constructed with RAxML under the GTR+G model of evolution which was applied to each codon partition. Statistical support was estimated with the GTRCAT model of rate heterogeneity and 1 000 rapid bootstrap (RBS) replicates.

Table S1. Individual data including: Individual number, Two letters Country ISO code, Locality (site or river name), Sea basin, River Catchment, River sub-catchment, River, Lattitude and Longitude in decimal format, Altitude (m), Site Acronym, Individual Acronym, Haplotype name, BIN-MOTUs, ABGD-MOTUs, bPTP-MOTUs, Genbank Accession number, BOLD Process ID, COI sequense Length in bp, Reference.

*NB: some Genbank accession numbers are identical as only a single haplotype was deposited associated with original publications. These sequences were ascribed to different individuals as based on data supplementary info available in each publication.*

Tab-S2. Primers used in the study. Primer names their direction and sequence, reverence and binding position according to reference COI full gene of *Gammarus fossarum* (GenBank accession: NC_034937.1).
