# Supplementary material for: Continental-scale patterns of hyper-cryptic diversity within the freshwater model taxon Gammarus fossarum (Crustacea, Amphipoda)
Source: Sci Rep. 2020 Oct 6;10:16536. doi: 10.1038/s41598-020-73739-0 (PMC7538970; doi:10.1038/s41598-020-73739-0)
Supplement: Supplementary file 4 — Supplementary Table S2. [file 41598_2020_73739_MOESM4_ESM.docx]

Table S2-Primers used in the study. Primer names their direction and sequence, reverence and binding position according to reference COI full gene of *Gammarus fossarum* (GenBank accession: NC_034937.1).

| Primer | Direction | Sequence 5'-3' | Reference | Position |
| --- | --- | --- | --- | --- |
| LCO1490 | Forward | GGTCAACAAATCATAAAGATATTGG | Folmer et al. (1994) | 17-41 |
| HCO2198 | Reverse | TAAACTTCAGGGTGACCAAAAAATCA | Folmer et al. (1994) | 700-725 |
| UCOIR | Forward | ACWAAYCAYAAAGAYATYGG | Costa et al. (2007) | 22-41 |
| UCOIF | Reverse | TAWACTTCDGGRTGRCCRAAAAAYCA | Costa et al. (2007) | 700-721 |
| LCO1490-JJ | Forward | CHACWAAYCATAAAGATATYGG | Astrin & Stuben (2008) | 20-41 |
| HCO2198- JJ | Reverse | AWACTTCVGGRTGVCCAAARAATCA | Astrin & Stuben (2008) | 700-724 |
| COIGrF | Forward | GCTAGHGCCGTAGGYACATC | Grabowski et al. (2017) | 22-41 |
| COIGrR2 | Reverse | RAATARGTGYTGGTACAGAATAGG | Grabowski et al. (2017) | 676-679 |

Folmer O, Black M, Hoeh W, Lutz R, & Vrijenhoek R (1994) DNA primers for amplification of mitochondrial cytochrome c oxidase subunit I from diverse metazoan invertebrates. Molecular Marine Biology and Biotechnology3(5):294-299.

Costa FO, et al. (2007) Biological identifications through DNA barcodes: the case of the Crustacea. Canadian Journal of Fisheries and Aquatic Sciences 64(2):272-295.

Astrin JJ & Stuben PE (2008) Phylogeny in cryptic weevils: molecules, morphology and new genera of western Palaearctic Cryptorhynchinae (Coleoptera : Curculionidae). Invertebrate Systematics 22(5):503-522.

Grabowski M, Mamos T, Bącela-Spychalska K, Rewicz T, & Wattier RA (2017) Neogene paleogeography provides context for understanding the origin and spatial distribution of cryptic diversity in a widespread Balkan freshwater amphipod. PeerJ 5:e3016.
